# An impact of superior vena cava isolation in non‐paroxysmal atrial fibrillation patients with low voltage areas

**DOI:** 10.1002/joa3.12552

**Published:** 2021-05-18

**Authors:** Takuya Omuro, Yasuhiro Yoshiga, Takeshi Ueyama, Akihiko Shimizu, Makoto Ono, Masakazu Fukuda, Takayoshi Kato, Hironori Ishiguchi, Shohei Fujii, Masahiro Hisaoka, Shigeki Kobayashi, Masafumi Yano

**Affiliations:** ^1^ Faculty of Health Sciences Yamaguchi University Graduate School of Medicine Ube Japan; ^2^ Department of Medicine and Clinical Science Yamaguchi University Graduate School of Medicine Ube Japan

**Keywords:** arrhythmia, atrial fibrillation, catheter ablation, electrophysiology, pulmonary vein isolation

## Abstract

**Background:**

This study aimed to investigate the correlation between left atrial low‐voltage areas (LVAs) and an arrhythmogenic superior vena cava (SVC) and the impact on the efficacy of an empiric SVC isolation (SVCI) along with a pulmonary vein isolation (PVI) of non‐paroxysmal atrial fibrillation (non‐PAF) with or without LVAs.

**Methods:**

We retrospectively enrolled 153 consecutive patients with non‐PAF who underwent a PVI alone (n = 51) or empiric PVI plus an SVCI (n = 102). Left atrial voltage maps were constructed during sinus rhythm to identify the LVAs (<0.5 mV). An arrhythmogenic SVC was defined as firing from the SVC and an SVC associated with the maintenance of AF‐like rapid SVC activity.

**Results:**

An arrhythmogenic SVC and LVAs were identified in 28% and 65% of patients with a PVI alone and 36% and 73% of patients with a PVI plus SVCI, respectively (*P* = .275 and *P* = .353). In the multivariate analysis a female gender, higher pulmonary artery systolic pressure (PAPs), and arrhythmogenic SVC were associated with the presence of LVAs. In the PVI plus SVCI strategy, there was no significant difference in the atrial tachyarrhythmia/AF‐free survival between the patients with and without LVAs after initial and multiple sessions (50% vs. 61%; *P* = .386, 73% vs. 79%; *P* = .530), however, differences were observed in the PVI alone group (27% vs. 61%; *P* = .018, 49% vs. 78%; *P* = .046).

**Conclusions:**

The presence of LVAs was associated with an arrhythmogenic SVC. An SVCI may have the potential to compensate for an impaired outcome after a PVI in non‐PAF patients with LVAs.

## Graphical abstract

The LVAs was associated with an arrhythmogenic SVC in non‐PAF patients. An SVCI compensated for an impaired outcome after PVI in non‐PAF patients with LVAs.

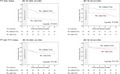

## 1 INTRODUCTION

Pulmonary vein (PV) isolation (PVI) is the cornerstone procedure for patients with all types of atrial fibrillation (AF).^1^ However, the outcome of a PVI alone is unsatisfactory in patients with non‐paroxysmal AF (PAF). To improve the outcome after catheter ablation of non‐PAF, various ablation strategies involving substrate modification have been devised. However, an empiric conventional substrate modification, such as left atrial linear ablation and complex fractionated atrial electrogram ablation, added to the PVI have been questioned as to whether they improve the outcome after ablation of persistent AF.^2^ The left atrial substrate parameters, such as the voltage and velocity, progress parallel to the progression of the AF type.^3^ It was reported that the presence of left atrial low‐voltage areas (LVAs) after the PVI has been shown to be a strong predictor of an AF recurrence, and LVAs could be targeted for substrate modification.^4^, ^5^, ^6^ However, LVA ablation in randomized controlled trials also failed to show any advantage over a conventional substrate modification.^7^ Therefore, searching for new additional ablation strategies beyond the PVI to improve the outcome after ablation of non‐PAF is necessary, especially in patients with an advanced arrhythmogenic substrate. The presence of LVAs is associated with not only a higher age, female gender, larger left atrium (LA), and persistent AF but also with sinoatrial node dysfunction, which suggests the spread of an arrhythmogenic substrate in the right atrium (RA).^8^ Although the superior vena cava (SVC) has been described as one of the most frequent non‐PV triggers ^9^, an empiric SVC isolation (SVCI) in addition to the PVI had limited effect after the initial procedure using a non‐contact force (CF) sensing catheter, especially in patients with non‐paroxysmal AF ^10^, ^11^, ^12^, ^13^. It has been reported that the durability of the PVI and SVCI using a non‐CF catheter is low ^14^, ^15^, and it is assumed that the use of a CF catheter has recently improved the durability of the PVI and SVCI. We hypothesized that strict thoracic vein isolation strategies using a CF sensing catheter, which contain a PVI and SVCI, determine the outcome after ablation in patients with non‐PAF. The first aim of this study was to investigate the correlation between the LVAs and arrhythmogenicity of the SVC. The second aim of this study was to test the hypothesis that a strict SVCI in addition to the PVI would improve the ablation outcomes in non‐PAF patients, with or without the presence of LVAs.

## 2 METHODS

### 2.1 Study population

This study was approved by the institution's ethics committee and followed the “Declaration of Helsinki” and the ethical standards of the responsible committee on human experimentation, and all patients underwent informed consent. Consecutive patients with non‐PAF, who underwent catheter ablation using a PVI alone or PVI plus SVCI strategy, were retrospectively enrolled into the current study. The PVI alone strategy was applied in the former half of the cases, and the PVI plus SVCI strategy was applied in the latter half of the cases. The PVI alone or PVI plus SVCI strategies were performed regardless of an arrhythmogenic SVC and the existence of LVAs. The follow‐up period was set at 18 months. Exclusion criteria were (a) an age <20 years, (b) prior surgery of the heart, lungs, or esophagus, (c) radiotherapy due to cancer in the thorax or previously receiving chemotherapy, and (d) a prior catheter ablation.

### 2.2 Electrophysiological study

Electrocardiogram (ECG)‐gated and contrast‐enhanced computed tomographic (CT) imaging was performed before the procedure. One multipolar 6Fr 20‐pole catheter (Be eat; Japan Lifeline, Tokyo, Japan) was positioned in the coronary sinus, which covered the high RA and SVC, via the right subclavian vein for pacing, continuous recording, and internal AF cardioversion throughout the procedure. The proximal electrodes enabled the mapping of the SVC during the entire procedure. An 8 Fr SoundStar ultrasound catheter (Biosense Webster, Diamond Bar, CA, USA) was inserted into the RA via the left femoral vein and anatomic mapping of the LA by a CartoSound module equipped with a CARTO3 system (Biosense Webster) was performed. Two 8.5 Fr long transseptal sheaths (SL1; St Jude Medical) were inserted into the LA using a modified Brockenbrough technique. Intracardiac echography (ICE) images were displayed through the CartoSound module using an Acuson X300PE echocardiography system (Siemens Medical Solutions). Each PV ostium was identified by selective venography and tagged on the electroanatomical map using a 3.5‐mm‐tip open‐irrigated CF sensing catheter (Navistar ThermocoolSmartTouch^TM^; Biosense Webster). A 20‐pole Lasso‐catheter (Biosense Webster) was placed within the superior PVs or within the superior branches of a common PV during the radiofrequency delivery.

### 2.3 Voltage mapping

Sinus rhythm was restored by external or internal cardioversion before the PVI, and then a voltage map was created 10 minutes later. If AF did not convert to sinus rhythm, the PVI was performed during AF. After the right or left PVI, external or internal cardioversion was repeatedly administered aiming at the restoration of sinus rhythm. In such a case, voltage mapping was performed after the PVI. Mapping of the LA was performed during sinus rhythm with a 20‐polar Pentaray catheter (Biosense Webster) using the CARTO mapping system and merged with CT integration (CARTOMERGE; Biosense Webster). Five hundred to 1000 LA mapping points per patient were carefully obtained. The band pass filter was set at 30 to 500 Hz. The bipolar peak‐to‐peak voltage at each acquired point was measured. LVAs were defined as those of <0.5 mV and covering >5% of the LA body surface area according to the published data.^3^, ^4^, ^5^, ^6^, ^7^ The CARTO system automatically calculated the surface area from the manually selected points. To exclude LVAs because of insufficient wall contact, the voltages were reconfirmed by the CF catheter introduced through the long sheath for the sites with apparent LVAs.

### 2.4 Ablation protocol during the initial procedure

All patients underwent a circumferential PVI using irrigated radiofrequency current and an integrated 3D image with CF guidance of more than 10 g. For 30 seconds at each point, irrigated radiofrequency energy was delivered using a target temperature of 43°C, maximum power of 20‐30 W, and infusion rate of 17 mL/min at the posterior, inferior, and roof aspects of both continuous circular lesions (CCLs). Radiofrequency energy with a maximum power of 30‐35 W and flow rate of 17‐30 mL/min was delivered to the anterior aspects of both CCLs. The procedural endpoint was defined as the absence or dissociation of all PV potentials, documented by the Lasso catheter, at least 60 minutes after the PVI during sinus rhythm.

One hundred‐two of the patients in this study underwent an SVCI in addition to the PVI. After confirmation of no PV reconduction, the mapping and ablation catheters were withdrawn back into the RA. The geometry of the RA was reconstructed, and the SVC‐RA junction was tagged on the geometry based on the SVC angiography. The circular catheter was placed just above the RA‐SVC junction. Segmental ablation targeting the earliest RA‐SVC junction was applied for the SVCI with CF guidance of more than 10 g. High output pacing (10 mA) was performed before the radiofrequency current delivery at the posterolateral aspect of the SVC. In such sites, ablation was avoided to prevent phrenic nerve injury. Irrigated radiofrequency energy was delivered for 20 seconds using a target temperature of 43°C, maximum power of 20‐25 W, and an infusion rate of 17 mL/min. A lower energy (20W) and lower CF (10‐15g) were applied on the lateral side as compared to the septal side of the SVC to prevent phrenic nerve palsy. An SVCI was characterized as the disappearance of the SVC potentials or the dissociation of the SVC potentials with RA activity. No patients underwent ablation of linear lesions, complex atrial electrograms, or ablation of non‐PV triggers. A cavotricuspid isthmus ablation was only performed in patients with a history of atrial flutter (AFL).

### 2.5 Identification of an arrhythmogenic superior vena cava

**FIGURE 1:**
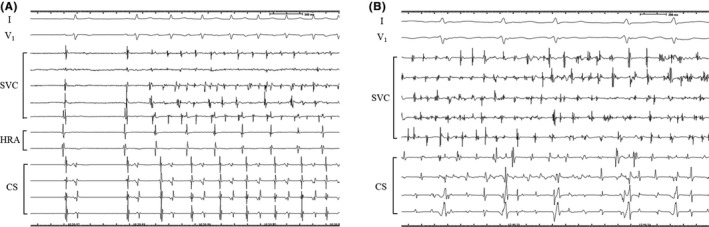
A circular mapping catheter is placed in the SVC. A, AF initiated from the SVC after cardioversion. Note that the SVC potentials are recorded following the RA potentials during sinus rhythm. B, At the beginning of the procedure, very rapid activity was observed inside the SVC during AF. CS; coronary sinus; HRA, high right atrium; SVC, superior vena cava

Mapping electrodes were placed in the SVC during the entire procedure (during AF, before and after cardioversion), and detailed mapping was performed when arrhythmogenicity of the SVC was suspected. During an isoproterenol infusion (1‐10 μg/min) after the PVI, a circular mapping catheter was placed to find if there was an arrhythmogenic SVC in the initial and repeat ablation procedures. A 20‐mg bolus of ATP was injected with the administration of the isoproterenol. An arrhythmogenic SVC was defined as firing from the SVC with or without triggering AF and an SVC associated with the maintenance of AF‐like rapid SVC activity (Figure 1).

### 2.6 Ablation protocol during repeat procedures

Repeated electrophysiological procedures were undertaken for any atrial tachyarrhythmias (ATs) lasting for 30 seconds, which included PAF, atrial tachycardia, and AFL and episodes of AF lasting for seven days (persistent AF). The initial strategy was an assessment of the PV reconduction during sinus rhythm after cardioversion, followed by the closure of all PV conduction gaps and an electrical re‐isolation. In patients with a PVI plus an SVCI strategy, SVC reconduction during sinus rhythm was also assessed, followed by the closure of all SVC conduction gaps and an electrical re‐isolation.

### 2.7 Post‐ablation management and clinical follow‐up

In all patients the anti‐arrhythmic drugs were discontinued after the procedure. For 3 days after the procedure, ECG monitoring was performed. The ECG and Holter ECG recordings were repeated at 1, 3, 6, 12, and 18 months after the procedure. If the patients complained of symptoms suggestive of an arrhythmia recurrence, an event monitor was provided. Long‐term success was defined as the absence of any sustained (>30 sec) ATs. Three‐month of blanking period was applied. The indication for a repeat procedure was left to the discretion of the physician.

### 2.8 Statistical analysis

Categorical variables are expressed as absolute and relative frequencies. Continuous variables are expressed as the mean ±SD or median and interquartile range as appropriate. Unpaired *t*‐tests, using a Wilcoxon rank‐sum test where appropriate, were used for the comparisons between groups. Categorical variables were compared using a Fisher's exact test. To test for predictors of LVAs we used a multivariate binary logistic regression. Baseline variables that were significant (*P* < .05) in the univariate analysis entered into the multivariate analysis. The event‐free rate was estimated by the Kaplan–Meier analysis using a log‐rank test. A two‐tailed probability value of <.05 was considered significant.

## 3 RESULTS

### 3.1 Patients characteristics, an arrhythmogenic SVC, and LA electro‐anatomical mapping

A total of 153 consecutive patients with non‐PAF, who underwent AF ablation by a PVI alone (n = 51) or PVI plus SVCI strategy (n = 102), was consecutively included in the study from April 2014 to March 2018. The baseline characteristics of the patient population are presented in Table 1. An arrhythmogenic SVC was identified in 14 of 51 (27.5%) patients with a PVI alone and 37 of 102 (36.3%) patients with a PVI plus SVCI (*P* = .275). LVAs were identified in 33 of 51 (64.7%) patients with a PVI alone and 74 of 102 (72.5%) patients with a PVI plus SVCI (*P* = .353). There was no significant difference in the baseline characteristics, an arrhythmogenic SVC, and identified LVAs between the PVI alone group and PVI plus SVCI group.

**TABLE 1:** Comparison between the PVI and PVI + SVCI group

	Al (n = 153)	PVI alone (n = 51)	PVI + SVCI (n = 102)	*P* value
Age, year	64.5 ± 9.8	62.7 ± 9.4	65.3 ± 9.8	.113
Male, n (%)	119 (77.8)	40 (78.4)	79 (77.5)	>.999
Body Mass Index	24.4 ± 3.4	24.5 ± 3.4	24.4 ± 3.4	.924
AF history, month (IQR)	8 (4‐23)	8 (4‐24)	8 (4‐17)	.375
Heart failure, n (%)	40 (26.1)	9 (17.6)	31(30.4)	.119
Hypertension, n (%)	88 (57.5)	26 (51.0)	62 (60.8)	.299
Diabetes mellitus, n (%)	31 (20.3)	10 (19.6)	21 (20.6)	>.999
Stroke, n(%)	10 (6.5)	4 (7.8)	6 (5.9)	.732
CHA2DS2 ‐ VASc score	2.2 ± 1.5	2.0 ± 1.7	2.4 ± 1.4	.144
Structural heart disease	30 (19.6)	12 (23.5)	18 (17.6)	.395
Valvular heart disease	8 (5.2)	1 (2.0)	7 (6.9)	.270
Ischemic heart disease	12 (7.8)	6 (11.8)	6 (5.9)	.216
Cardiomyopathy	8 (5.2)	5 (9.8)	3 (2.9)	.118
Congenital heart disease	2 (1.3)	0 (0.0)	2 (2.0)	.553
Echocardiographic findings				
LVEF, %	61.1 ± 11.4	60.9 ± 11.3	61.2 ± 11.5	.881
LAD, mm	45.0 ± 7.5	45.2 ± 6.3	44.8 ± 8.0	.784
LAVI, ml/m2	51.4 ± 16.2	49.2 ± 15.8	52.5 ± 16.4	.239
E, cm/s	80.8 ± 20.3	82.2 ± 14.9	80.1 ± 22.5	.550
E wave DcT, ms	187.1 ± 42.7	180.3 ± 35.6	190.5 ± 45.6	.166
e', cm/s	9.0 ± 2.2	9.0 ± 2.4	9.0 ± 2.2	.996
E/e'	9.7 ± 4.2	10.0 ± 4.3	9.5 ± 4.1	.479
RV‐RA PG, mmHg	21.7 ± 4.6	21.4 ± 4.8	21.8 ± 4.5	.583
Estimated PAPs, mmHg	27.0 ± 4.5	26.8 ± 4.8	27.1 ± 4.4	.726
SVC sleeve length (mm)	34.5 ± 7.2	33.9 ± 5.8	34.7 ± 7.9	.525
Arrhythmogenic SVC, n (%)	51 (33.3)	14 (27.5)	37 (36.3)	.275
LVAs ≥ 5%, n (%)	107 (69.9)	33 (64.7)	74 (72.5)	.353

Abbreviations: DcT, deceleration time; IQR, interquartile range; LAD, left atrial dimension; LAVI, left atrial volume index; LVAs, low voltage areas; LVEF, left ventricular ejection fraction; PAPs, systolic pulmonary artery pressure; PG, pressure gradient; PVI, plumonary vein isolation; RA, right atrium; RV, right ventricle; SVCI, superior vena cava isolation

**FIGURE 2:**
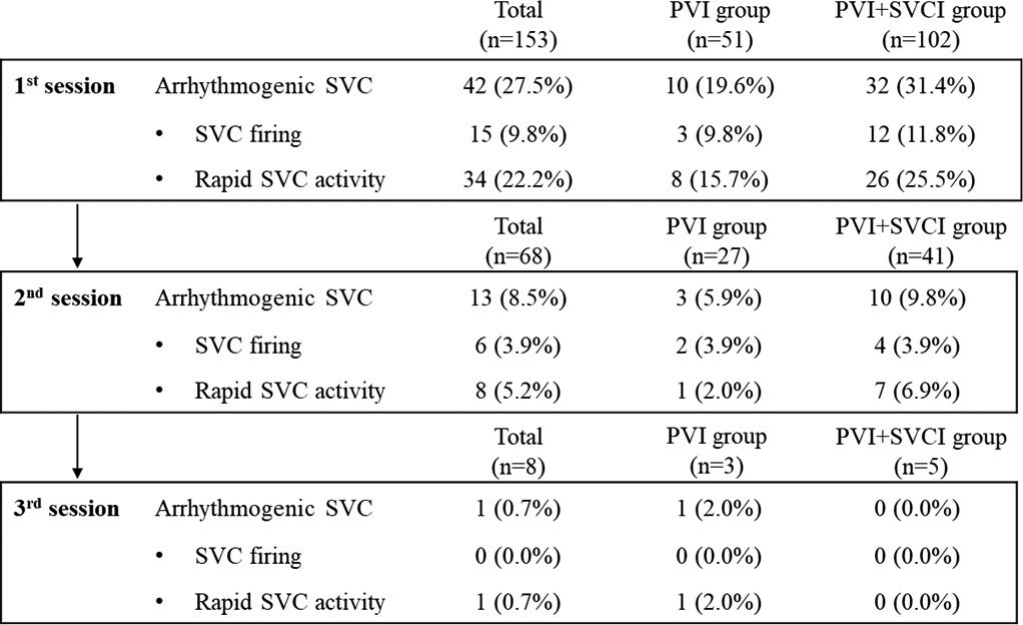
Flowchart demonstrating the incidence of an arrhythmogenic SVC. PVI, pulmonary vein isolation; SVC, superior vena cava; SVCI, superior vena cava isolation

An arrhythmogenic SVC was identified in 42 of 153 (27.5%), 13 of 68 (19.1%), and 1 of 8 (12.5%) patients in the first, second, and third sessions, respectively. An SVC firing, SVC associated with the maintenance of AF, and both were identified in 21 (13.7%), 38 (24.8%), and 8 (5.2%) patients, respectively, in the total sessions. There was no significant difference in the total number of arrhythmogenic SVCs, SVC firing, and SVCs associated with the maintenance of AF in each session between the PVI alone group and PVI plus SVCI group (Figure 2).

**FIGURE 3:**
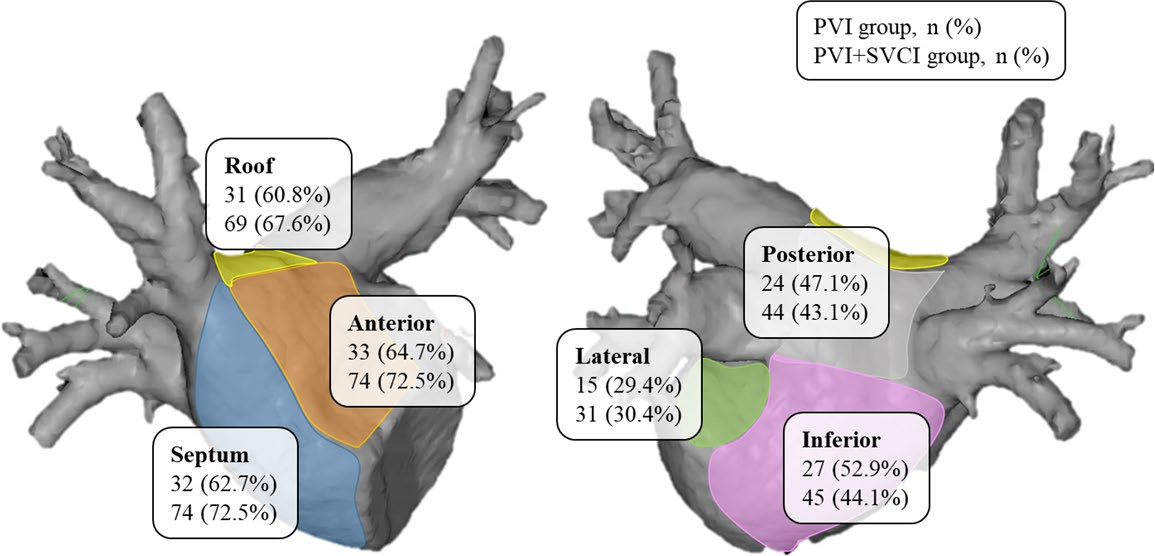
Left atrial distribution of LVAs. Upper line: PVI alone group, Lower line: PVI plus SVCI group. PVI, pulmonary vein isolation; SVCI, superior vena cava isolation

An LA voltage map was created during sinus rhythm with mean sampling points of 717 ± 328 in the PVI alone group and 741 ± 357 in the PVI plus SVCI group. LVAs existed in 33 (64.7%) patients with an area of 8.4 (IQR; 1.3‐19.3) cm^2^, occupying 11.6 (IQR; 2.1‐26.6) % in the PVI alone group and in 74 (72.5%) patients with an area of 9.5 (IQR; 3.3‐19.4) cm^2^, occupying 13.3 (IQR; 4.5‐25.0) % in the PVI plus SVCI group. The distribution and area of the LVAs were similar between the PVI alone and PVI plus SVCI group (Figure 3).

### 3.2 Predictors of LVAS

The comparison of the patient characteristics between the patients with and without LVAs is shown in Table 2. LVAs were more frequently found in the patients with a higher age (66.0 ± 9.2 vs. 60.9 ± 10.2 years; *P* = .003), female gender (27.1% vs 10.9%; *P* = .033), higher CHA2DS2‐VASc score (2.5 ± 1.5 vs. 1.7 ± 1.3; *P* = .003), higher E/e’ (10.2 ± 4.6 vs. 8.5 ± 2.8; *P* = .021), higher right ventricle (RV)‐RA pressure gradient (PG) (22.8 ± 4.1 vs. 19.2 ± 4.5 mmHg; *P* < .001), higher estimated systolic pulmonary artery pressure (PAPs) (27.9 ± 4.1 vs. 24.8 ± 4.7 mmHg; *P* < .001), and arrhythmogenic SVC (41.1% vs 15.2%; *P* = .002).

**TABLE 2:** The comparison of the patient characteristics between patients with and without LVAs

	Pts. witout LVAs (n = 46)	Pts. with LVAs (n = 107)	*P* value
Age, year	60.9 ± 10.2	66.0 ± 9.2	.003
Female, n (%)	5 (10.9)	29 (27.1)	.033
Body Mass Index	24.4 ± 3.4	24.4 ± 3.4	.922
AF history, month (IQR)	10 (6‐26)	7 (3‐17)	.980
Heart failure, n (%)	11 (23.9)	29 (27.1)	.841
Hypertension, n (%)	23 (50.0)	65 (60.7)	.285
Diabetes mellitus, n (%)	8 (17.4)	23 (21.5)	.664
Stroke, n(%)	2 (4.3)	8 (7.5)	.724
CHA2DS2 ‐ VASc score	1.7 ± 1.3	2.5 ± 1.5	.003
Structural heart disease	7 (15.2)	23 (21.5)	.506
Valvular heart disease	2 (4.3)	7 (6.5)	.725
Ischemic heart disease	2 (4.3)	10 (9.3)	.512
Cardiomyopathy	3 (6.5)	5 (4.7)	.697
Congenital heart disease	0 (0.0)	2 (1.9)	>.999
Echocardiographic findings			
LVEF, %	59.5 ± 11.0	61.8 ± 11.5	.244
LAD, mm	43.9 ± 5.8	45.4 ± 8.1	.247
LAVI, ml/m2	48.1 ± 14.3	52.8 ± 16.8	.099
E, cm/s	75.9 ± 18.2	82.9 ± 20.8	.049
E wave DcT, ms	186.6 ± 36.3	187.3 ± 45.3	.925
e', cm/s	9.4 ± 2.2	8.9 ± 2.3	.224
E/e'	8.5 ± 2.8	10.2 ± 4.6	.021
RV‐RA PG, mmHg	19.2 ± 4.5	22.8 ± 4.1	<.001
estimated PAPs, mmHg	24.8 ± 4.7	27.9 ± 4.1	<.001
SVC sleeve length (mm)	33.0 ± 8.2	35.1 ± 6.7	.104
Arrhythmogenic SVC, n (%)	7 (15.2)	44 (41.1)	.002

Abbreviations: DcT, deceleration time; IQR, interquartile range; LAD, left atrial dimension; LAVI, left atrial volume index; LVAs, low voltage areas; LVEF, left ventricular ejection fraction; PAPs, systolic pulmonary artery pressure; PG, pressure gradient; RA, right atrium; RV, right ventricle; SVC, superior vena cava

In the univariate analysis the following variables predicted the presence of LVAs; age, female gender, CHA2DS2‐VASc score, E/e’, estimated PAPs, and arrhythmogenic SVC. In the multivariate analysis a female gender (OR 4.023, 95% CI 1.049‐15.425, *P* = .042), higher PAPs (OR 1.122, 95% CI 1.194‐1.243, *P* = .028), and arrhythmogenic SVC (OR 3.134, 95% CI 1.194‐8.228, *P* = .020) remained statistically significant (Table 3).

**TABLE 3:** Multivariate analysis of the predictors of LVAs in patients with non‐PAF

	Uni variate			Multi variate		
	OR	95% CI	*P* value	OR	95% CI	*P* value
Age, year	1.056	1.017‐1.096	.004	1.025	0.968‐1.804	.397
Female, n (%)	3.049	1.098‐8.468	.032	4.023	1.049‐15.425	.042
Body Mass Index	1.006	0.908‐1.113	.914			
AF history, month (IQR)	1.000	0.987‐1.013	.980			
Heart failure, n (%)	1.183	0.531‐2.634	.681			
Hypertension, n (%)	1.548	0.772‐3.104	.219			
Diabetes mellitus, n (%)	1.301	0.534‐3.171	.563			
Stroke, n(%)	1.778	0.363‐8.715	.478			
CHA2DS2–VASc score	1.504	1.143‐1.979	.004	1.113	0.750‐1.651	.595
Structural heart disease	1.526	0.603‐3.856	.372			
Echocardiographic findings						
LVEF, %	1.014	0.984‐1.046	.352			
LAD, mm	1.031	0.975‐1.090	.290			
LAVI, ml/m^2^	1.020	0.995‐1.046	.113			
E wave, cm/s	1.020	1.000‐1.041	.050			
E wave DcT, ms	1.002	0.993‐1.010	.723			
e', cm/s	0.909	0.777‐1.063	.232			
E/e'	1.147	1.017‐1.293	.025	1.053	1.013‐1.243	.439
estimated PAPs, mmHg	1.188	1.085‐1.300	<.001	1.122	1.194‐1.243	.028
SVC sleeve length (mm)	1.04	0.992‐1.092	.106			
Arrhythmogenic SVC, n (%)	3.891	1.595‐9.494	.003	3.134	1.194‐8.228	.020

Abbreviations: DcT, deceleration time; IQR, interquartile range; LAD, left atrial dimension; LAVI, left atrial volume index; LVAs, low voltage areas; LVEF, left ventricular ejection fraction; OR, odds ratio; PAF, paroxysmal atrial fibrillation; PAPs, systolic pulmonary artery pressure; PG, pressure gradient; SVC, superior vena cava

### 3.3 Clinical outcome analysis: the comparison between the patients with and without LVAS

**FIGURE 4:**
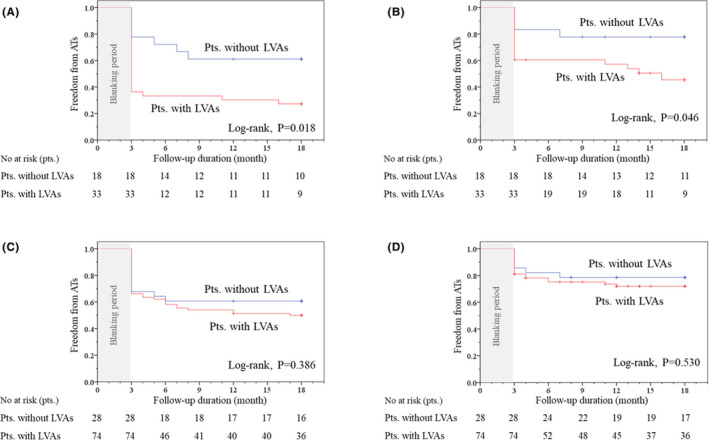
Comparison of the freedom from ATs after the initial and last procedure between the patients with and without LVAs. The graph shows the Kaplan‐Meier estimates of the freedom from documented ATs after the initial (A) and last (B) procedure in the patients with the PVI alone strategy and after the initial (C) and last (D) procedure in the patients with the PVI plus SVCI strategy. ATs, atrial tachyarrhythmias; LVAs, low voltage areas; Pts, patients

The PVs in all 153 patients and the SVC in all 102 patients with a PVI plus SVCI strategy were completely isolated during the first procedure. In patients with a PVI alone strategy, the PV conduction was found to have recovered in 20 of 27 (74.1%) patients during the second procedure and none of 3 patients during the third procedure. Nine patients without any recovered PV conduction were judged to have recurrences after achieving the strategic endpoint. In patients with a PVI plus SVCI strategy, the PV conduction was found to have recovered in 28 of 41 (68.3%) patients during the second procedure and none of 5 patients during the third procedure. The SVC conduction was found to have recovered in 34 of 41 (82.9%) patients during the second procedure and 2 of 5 (40.0%) patients during the third procedure. Three patients without any recovered PVs or SVC conduction were judged to have recurrences after achieving the strategic endpoint. All conduction gaps were successfully closed with a minimal number of irrigated radiofrequency current applications during the repeat procedures in both groups. In one patient each with a PVI alone and a PVI plus SVCI strategy, respectively, no conduction gaps were found and they underwent a cavo‐tricuspid isthmus ablation for recurrent AFL. A total of 81 and 148 procedures were performed in 51 and 102 patients for the strategies with a PVI alone and PVI plus an SVCI. No major complications were observed in any of the groups. Vascular access complications were observed in one patient with a PVI alone strategy. Phrenic nerve palsy was not observed in the PVI plus SVCI strategy group. No patients were lost to follow‐up. There were significant differences in the maintenance of sinus rhythm between the patients with and without LVAs after the initial (27.3%, 9/33 patients vs. 61.1%, 11/18 patients: *P* = .018) and multiple procedures (48.5%, 16/33 patients vs. 77.8%, 14/18 patients: *P* = .046) in the patients with a PVI alone strategy (Figure 4A,B). However, in the patients with a PVI plus SVCI strategy, a difference in the maintenance of sinus rhythm could not be found between the patients with and without LVAs after the initial (50.0%, 37/74 patients vs. 60.7%, 17/28 patients: *P* = .386) and multiple procedures (73.0%, 54/74 patients vs. 78.6%, 22/28 patients: *P* = .530) (Figure 4C,D).

## 4 DISCUSSION

Our study of patients undergoing catheter ablation using a PVI alone or PVI plus SVCI strategy for non‐PAF produced the following results: (a) the presence of LVAs was associated with a female sex, the estimated PAPs, and an arrhythmogenic SVC; (b) in the PVI plus SVCI strategy, there were no significant differences in the ATs recurrence between the patients with and without LVAs, however, a difference was observed for the PVI alone strategy.

### 4.1 Low voltage areas as the substrate of AF

Low‐voltage electrograms are mainly due to atrial fibrosis. AF also promotes atrial fibrosis caused by tissue remodeling and perpetuates the maintenance of AF. Electrophysiologically, atrial fibrosis produces lower amplitude electrograms, fractionated electrograms, and a conduction heterogeneity that can be identified using electroanatomic mapping during sinus rhythm. Therefore, fibrotic remodeling tissue acts as an AF substrate because it exhibits slow conduction and a short action potential duration, which facilitates reentry.^16^, ^17^ It is reported that the presence of LVAs is associated with a higher age, female gender, larger LA, sinoatrial node dysfunction, and persistent AF.^8^ LVAs are observed in patients with persistent AF, with a prevalence of 35%‐84%.^5^, ^6^, ^7^, ^8^, ^18^, ^19^ In this study, LVAs were observed in 107 (69.9%) of 153 non‐PAF patients, and in the patients with a higher age, female gender, higher CHA_2_DS_2_‐VASc scores, higher E/e’, higher estimated PAPs, and arrhythmogenic SVC. This was in line with the prior studies. A previous report and our results suggest that an arrhythmogenic substrate in patients with LVAs spreads to not only the LA but also the RA. LVAs most likely act as arrhythmogenic substrates, promoting more persistent AF by slowing the electrical conduction and sustaining fibrillatory conduction. Therefore, residual triggers after thoracic vein isolation may induce AF easily in patients with LVAs compared to those without LVAs.

### 4.2 Superior vena cava as the arrhythmogenic origin of AF

The SVC, one of the most common sites of non‐PV foci, has been established as an important source of AF.^9^ However, the incidence of AF originating from the SVC is unknown. In general, an arrhythmogenic SVC is defined as an SVC triggering AF, SVC initiating AF, and/or SVC associated with the maintenance of AF. An isoproterenol infusion, ATP bolus injection, and cardioversion of reinitiated AF are the techniques for the provocation of non‐PV triggers. It has been reported that arrhythmogenic SVCs are identified in 2.2% to 19.4% of PAF patients using various provocation techniques.^20^, ^21^, ^22^ An arrhythmogenic SVC is rarely identified in patients with persistent or long‐standing persistent AF.^23^, ^24^ The mechanism why the frequency of an SVC origin of AF is lower in non‐PAF than PAF is unclear. In this study, an arrhythmogenic SVC was identified in 51 cases (33.3%) by observation during AF, cardioversion and provocation maneuvers with an isoproterenol infusion, and an ATP bolus injection. The discrepancy in the incidence of arrhythmogenic SVCs between the several reports and our results may be due to the different definition of an arrhythmogenic SVC and provocation maneuvers. We defined an arrhythmogenic SVC as not only SVC firing triggering AF but also SVC firing not triggering AF and rapid SVC activity during AF. In fact, SVC firing and rapid SVC activity during AF were observed in 13.7% and 24.8% in this study, respectively.

### 4.3 The effects of a superior vena CAVA isolation in non‐PAF patients with low voltage areas

The SVC has been described as one of the most frequent non‐PV triggers.^9^ However, an empiric SVC isolation (SVCI) in addition to the PVI had a limited effect, especially in patients with non‐PAF.^10^, ^11^, ^12^, ^13^ The differences in the efficacy of the SVCI depending on the arrhythmogenic substrate of AF, which is suggested in this study, may explain the reason why the effects of an empirical SVCI are controversial.

In general, the procedure added to the PVI for AF with LVAs is substrate modification for LVAs. Some papers reported that a regional LVA ablation in addition to the PVI significantly improved the long‐term success rate in patients with LVAs. The outcome of the patients with a PVI plus substrate modification for LVAs was comparable to that of the patients without LVAs.^4^, ^6^ In our study, an SVCI in addition to the PVI had a similar effect on the ATs recurrence for the substrate modification of LVAs. It is reported that SVC ectopic beats initiating PAF were observed more often in a female gender.^25^ Since LVAs are more frequently associated with a female gender, patients with LVAs may have an SVC‐origin of AF more frequently. Recently, it was reported that the strategy of empiric SVCI plus PVI in recurred cases after first procedure for PAF, who are speculated to have more arrhythmogenic substrate, improved ATs recurrence free rate without increasing procedural time or complications.^26^ Therefore, empiric SVCI may be more effective for the patients difficult to control by PVI. Furthermore, in this study, the patients with LVAs had a higher E/e’ and estimated PAPs than the patients without LVAs, which suggested the hemodynamic load on the RA and LA, and resulted in the correlation between the LVAs and arrhythmogenic SVC. Therefore, patients with LVAs may be required to undergo treatment for the RA and SVC. AF occurs due to PV and non‐PV triggers and is maintained on the basis of an arrhythmogenic atrial substrate. Both triggers and an AF substrate are required for the generation and maintenance of AF. Therefore, a strict thoracic vein isolation may be as effective in suppressing AF as an aggressive arrhythmogenic substrate modification.

### 4.4 Safety and limitations of a contact force guided empiric superior vena CAVA isolation

Right phrenic nerve injury is a major concern during an SVCI. In addition, a high CF may increase the risk of phrenic nerve injury. In this study, no phrenic nerve injury was detected after the SVCI. Therefore, we could not comment on what the predictors of phrenic nerve palsy were in our case series. Applying a lower energy and CF on the lateral side and avoiding applications at sites with phrenic nerve capture during high output pacing might protect against phrenic nerve injury. On the other hand, such an ablation setting might lead to a high RA‐SVC reconnection rate in patients with repeat procedures. Recently, the ablation index has been developed as a novel marker of the lesion quality incorporating the CF, time, and power in a weighted formula, and has been shown to improve the one‐year outcome and prevent reconnections by using an appropriate ablation index value.^27^ Understanding the appropriate ablation settings to suppress reconnections after an SVCI without complications is desired to improve the outcome after the initial procedure with a PVI plus SVCI strategy.

## 5 STUDY LIMITATION

This study has several limitations. First, this is a retrospective single‐center study with a relatively small sample size. Thus, larger multicenter prospective studies are needed to confirm our findings. Second, the recurrence rate of ATs/AF might have been underestimated because asymptomatic ATs/AF episode might have been undetected by using 24‐hour ambulatory monitoring as compared with implantable loop recorders. Third, the confidence module of the CARTO 3 system was not used in this case series because the confidence module had not been introduced at the time of the first procedure. Poor contact of the mapping catheter was one of the most important limitations for detecting the accurate LVAs. Thus, our findings need to be confirmed using a new mapping system in order to detect more accurate LVAs.

## 6 CONCLUSIONS

The presence of LVAs was associated with a female gender, the estimated PAPs, and an arrhythmogenic SVC. In the PVI plus SVCI strategy, pre‐existence of LVAs as detected by LA voltage mapping has not been shown to be a predictor of an arrhythmia recurrence after an AF catheter ablation. These findings suggest that a strict thoracic vein isolation is the one of the options for catheter ablation in non‐PAF patients with LVAs.

## CONFLICT OF INTEREST

The authors declare that there is no conflict of interest.

## Data Availability

The data sets analyzed in this study are available from the corresponding author on reasonable request.
